# HIV-1 envelope trimer elicits higher neutralizing antibody responses than monomeric gp120

**DOI:** 10.1186/1742-4690-9-S2-O62

**Published:** 2012-09-13

**Authors:** JM Kovacs, JP Nkolola, H Peng, A Cheung, J Perry, CA Miller, MS Seaman, D Barouch, B Chen

**Affiliations:** 1 Harvard Medical School and Children's Hospital Boston, Boston, MA, USA; 2 Beth Israel Deaconess Medical Center and Ragon Institute, Boston, MA, USA; 3 Children's Hospital Boston, Boston, MA, USA; 4 Beth Israel Deaconess Medical Center, Boston, MA, USA

## Background

HIV-1 envelope glycoprotein is the primary target for HIV-1-specific antibodies. The native HIV-1 envelope spike on the virion surface is a trimer, but trimeric gp140 and monomeric gp120 are currently believed to induce comparable immune responses. Indeed, most studies on the immunogenicity of HIV-1 envelope oligomers have revealed only marginal improvement over monomers. We report here that stable and homogenous envelope trimers with characteristics expected for the native viral spikes are substantially superior at eliciting neutralizing antibodies in guinea pigs.

## Methods

Stable envelope gp140 trimer derived from clinical isolate sequences were stabilized with the T4-fibritin C-terminal trimerization tag and produced in stablely transfected 293T cells. Characterization of Env trimers was performed by Western blotting, size-exclusion chromatography (SEC), analytical-ultra centrifugation (AUC), multi-angle light scattering (MALS) and surface plasmon resonance (SPR). Guinea pigs were immunized six times with 100 µg of protein trimer or monomer in CpG/Emulsigen adjuvants. Antibody responses were determined by ELISA and TZM.bl neutralizing antibody assays.

## Results

Homogeneous trimer and monomer preparations exhibited high purity as measured by SEC and SDS-PAGE. AUC and MALS analyses revealed expected molecular weight for both trimer and monomer. SPR analyses revealed expected binding with CD4 and multiple broadly neutralizing antibodies. These trimers have markedly different antigenic properties than those of monomeric gp120s derived from the same sequences. They induce potent, cross-clade neutralizing antibody responses with titers substantially higher than those elicited by the corresponding gp120 monomers for a diverse set of both tier 1 and tier 2 viruses.

## Conclusion

We have demonstrated the importance of generating high-quality envelope trimers for antigenic and immunogenic studies; furthermore these results highlight the immunologic differences between monomers and high-quality envelope trimers, illustrating important implications for HIV-1 vaccine development and immunogen selection in large clinical trials.

